# Current era outcomes of pulmonary atresia with ventricular septal defect: A single center cohort in Thailand

**DOI:** 10.1038/s41598-020-61879-2

**Published:** 2020-03-20

**Authors:** Kanthalas Lertsakulpiriya, Chodchanok Vijarnsorn, Prakul Chanthong, Paweena Chungsomprasong, Supaluck Kanjanauthai, Kritvikrom Durongpisitkul, Jarupim Soongswang, Thaworn Subtaweesin, Somchai Sriyoschati

**Affiliations:** 1 Department of Pediatrics, Faculty of Medicine Siriraj Hospital, Mahidol University, Bangkok, Thailand; 2 Department of Surgery, Faculty of Medicine Siriraj Hospital, Mahidol University, Bangkok, Thailand

**Keywords:** Interventional cardiology, Congenital heart defects

## Abstract

Pulmonary atresia with ventricular septal defect (PA/VSD) is a complex cyanotic congenital heart disease with a wide-range of presentations and treatment strategies, depending on the source of pulmonary circulation, anatomy of pulmonary arteries (PAs), and major aortopulmonary collateral arteries (MAPCAs). Data about the outcomes in developing countries is scarce. We therefore conducted a retrospective study to assess survival rates and mortality risks of 90 children with PA/VSD at Siriraj Hospital, Thailand during 2005–2016. Patients with single ventricle were excluded. Survival and mortality risks were analyzed at the end of 2018. The median age of diagnosis was 0.5 (0–13.8) years. The patients’ PAs were categorized into four groups: 1) PA/VSD with confluent PAs (n = 40), 2) PA/VSD with confluent PAs and MAPCAs (n = 21), 3) PA/VSD with non-confluent PAs and MAPCAs (n = 12), and 4) PA/VSD with small native PAs and MAPCAs (n = 17). Of the 88 patients who underwent operations, 32 patients had complete repair at 8.4 ± 4.6 years old. During the follow-up [median time of 5.7 years (7 days-13.6 years)], 17 patients (18.9%) died. The survival rates at 1, 5, and 10 years of age were 95%, 83.7%, and 79.6%, respectively. Significant mortality risks were the presence of associated anomalies and non-confluent PAs.

## Introduction

Pulmonary atresia with ventricular septal defect (PA/VSD) is a complex congenital cyanotic heart disease that can lead to various clinical manifestations according to the individual central pulmonary artery anatomy and pulmonary distribution^1,2^. The intracardiac morphology of PA/VSD resembles the extreme form of tetralogy of Fallot, which results from an anterior deviation of the conal septum with the atretic right ventricular outflow in embryogenesis. Patients with the most simplistic form of PA/VSD, with central pulmonary artery and a ductus-dependent pulmonary circulation, often present with cyanosis in neonate, requiring an intervention such as prostaglandin administration or aortopulmonary shunt. A goal of the surgical treatment is the relatively straight-forward complete repair with closure of VSD and constructed continuity from right ventricle to the central pulmonary artery^1,3^. In a more complex subgroup of patients, postulated to have an interruption of blood flow through the ductus arteriosus into the pulmonary artery in utero, leading to inadequate segmental lung perfusion and major aortopulmonary collateral arteries (MAPCAs), form the so called PA/VSD with MAPCAs^4,5^. The central pulmonary artery may be diminutive, with a seagull-like appearance, or absent in the extremely severe patients. The presentations of the spectrum of cases are wide-ranging clinical variations from minimal cyanosis with congestive failure in the largely pulmonary-supplied blood flow by MAPCAs to progressive cyanosis in small-sized MAPCAs and pulmonary distribution. Strategies for treating PA/VSD with MAPCAs of single-stage and multi-stage repairs such as unifocalization have been long debated with regards to size and distribution of intrapulmonary arteries and the anatomy of the MAPCAs^6,7^. The prevailing treatment strategy is primarily to reconnect the pulmonary artery tree (unifocalization) and create an appropriate source of pulmonary blood supply to optimize the segmental lung perfusion and avoid segmental pulmonary hypertension. This is followed by a staged, complete repair comprised of reconnecting the pulmonary artery tree to the right ventricle and closure of VSD. Early cohorts have reported poor, long-term outcomes of PA/VSD in relation to the presence of MAPCAs, with 10- and 20-year survival rates of 50% and 20%, respectively^8^. Palliative treatment has been suggested for some patients with diminutive pulmonary beds; however, a combination of catheter intervention and surgical operation for pulmonary rehabilitation may allow some patients to achieve total correction with VSD closure^8,9^. Modern survival rates have been reported to be better than those of earlier study periods^10,11^.

In Thailand, the initial medical treatment for this lesion is usually initiated at a provincial hospital, followed by referral of patients to advanced cardiac centers according to the complexity of the disease and the repair strategies. Therefore, studies of the natural history and outcomes of PA/VSD in this country are limited. Siriraj Hospital is one of a few referral centers that offered stabilization and treatments for children with PA/VSD. Currently, some patients with complex PA/VSD at the medical center reach adulthood. The present study evaluates survival rates and mortality risks of children who were diagnosed with PA/VSD at Siriraj Hospital in the last decade.

## Results

### Demographics

**Figure 1:**
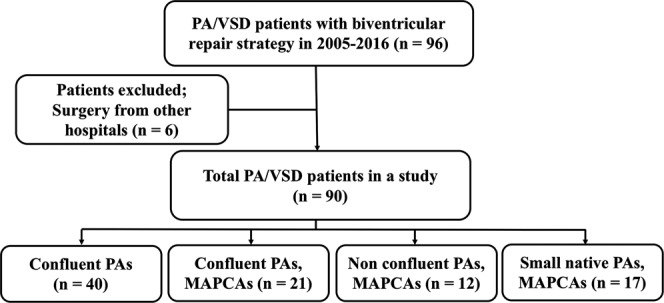
Flow of the study (n = 90). PA/VSD, pulmonary atresia with ventricular septal defect. PAs: pulmonary arteries; MAPCAs: major aortopulmonary collateral arteries.

A total of 90 patients with PA/VSD (44.4% were male) were included in the study cohort. The flow of the study is shown in Fig. 1. Median age of referral and diagnosis at our center was six months of age. Twenty-two patients (24.4%) were identified with other associated anomalies; for example, VACTERL (vertebral defect, anal atresia, cardiac anomalies, trachea-esophageal fistula, renal anomalies, and limb abnormalities), duodenal atresia, sensori-neural hearing loss, renal agenesis, global developmental delay, polydactyle, esotropia, and microcephaly. With regards to the patients’ pulmonary arteries, the patients were categorized into four groups: 1) PA/VSD with confluent PAs (n = 40), 2) PA/VSD, confluent PAs, MAPCAs (n = 21), 3) PA/VSD, non-confluent PAs, MAPCAs (n = 12), and 4) PA/VSD, small native PAs, MAPCAs (n = 17) (Fig. 1). Thus, complex PA/VSD, defined as pulmonary blood supply derived exclusively from MAPCAs, not a ductus arteriosus, were identified in one-third of the patients. Twenty-five patients had confluent pulmonary arteries and cardiac catheterization was performed prior to the cardiac operation. The ratio of right and left pulmonary artery size to abdominal aorta (McGoon ratio) was 1.55 ± 0.51. One premature infant with PA/VSD, confluent PAs supplied by PDA, had been referred from another hospital postnatally to repair a tracheoesophageal fistula (TEF). This patient died immediately after the TEF operation and had no cardiac operation for PA/VSD. The patients’ baseline characteristics are summarized in Table 1.

**Table 1:** Patients’ characteristics (n = 90).

Variables	
Male Gender	40 (44.4%)
Age at diagnosis (years)	0.5 (0–13.8)
Birth weight <2500 grams	27 (30%)
Presence of Genetic disorders	5 (5.5%)
Down syndrome	1 (1.1%)
22q11deletion	4 (4.4%)
Presence of associated anomalies	22 (24.4%)
Oxygen saturation at first presentation <80%	36 (40%)
Presence of MAPCAs	50 (56%)
Presence of pulmonary non-confluence (group 3)	12 (13%)

Data represented by median (range) and number (%).

MAPCAs, major aorto-pulmonary collateral arteries.

### Surgical management

Of the 90 patients, 2 had not undergone a cardiac operation in the cohort; 1 died prior to cardiac surgery and 1 was on a surgical list for unifocalization. Overall, 88 patients underwent 155 surgical operations at a median age at recent follow-up of 6.7 years old (7 days-26 years). The first operations were perfomed at a median age of 1.2 years of age (1 day-14.1 years). Of the 88 patients who underwent cardiac surgery, 32 patients achieved the complete staged surgical operation and total repair (see Supplementary Table S1). The mean age of complete repair was 8.4 ± 4.6 years. No VSD was left open at the stage of complete repair. Complete repair with the transanular pericardial monocusp technique was performed in one patient. Most of the patients underwent a repair with right ventricular to pulmonary artery conduit; aortic homograft (n = 2; 6.3%), pulmonic homograft (n = 5; 15.6%), bovine jugular valved conduit; Contegra (n = 22; 68.7%), and stentless porcine valve (n = 2; 6.3%). Size of conduit varied from 14 mm to 25 mm. Average conduit diameter was 18.3 ± 2.8 mm. Patients under the age of 8 years used significantly smaller conduit sizes compared to those used by the older patients (16.9 ± 2.6 mm and 19.4 ± 2.5 mm, respectively; *p*-value 0.01).

Of the 40 patients in group 1, 39 underwent 65 cardiac surgical interventions. Of these 39 patients, the first-stage operations were mainly aortopulmonary shunt: 29 right modified Blalock Taussig shunt (RMBTS), 7 left modified Blalock Taussig shunt (LMBTS), 1 central shunt. Shunt thrombosis was found in 1 patient (2.7%), requiring an additional central shunt. Two patients underwent total correction at their first stage: one was a 1.2 year-old boy who had a 14 mm Contegra bovine valve conduit and the other one was a 2.5 year-old girl who had a transannular pericardial monocusp repair. Overall, 15 patients in this group achieved total correction at the mean age of 5.4 ± 3.2 years. At the median time of follow-up (5 years), 1 patient required surgical left pulmonary arterioplasty postoperatively.

In group 2 (n = 21), 20 patients had 39 surgical operations. One patient, a 4-year-old girl, was on a waiting list for left unifocalization. Of the 20 patients who had surgical interventions, 11 achieved the stage of total correction following staged unifocalization or aortopulmonary shunt with or without ligation of MAPCAs. The mean age at total correction was 10.8 ± 4.5 years. During the median time of follow up (7 years), no redo pulmonary valve replacement or surgical arterioplasty was required.

In group 3 (n = 12), all patients had undergone staged surgical operations, starting with unifocalization plus Blalock Taussig shunt with or without constructed pulmonary artery by composited goretex graft. No midline unifocalization was performed at our medical center. The median age for the first staged surgery was 2.4 years (0.07–10.9 years). Overall, 24 operations were performed. Of the 12 patients, 4 achieved complete repair. In a median time of follow-up for this group (5 years), surgical pulmonary arterioplasty and thrombectomy were performed at 3 months and 2 years, respectively, in 2 patients after complete repair.

In group 4 (n = 17), 27 surgical operations were performed. The first staged palliation comprised of unifocalization and then Blalock Taussig shunt with or without constructed pulmonary artery by composite Goretex graft (n = 12), central shunt or palliative Blalock Taussig shunt (n = 4) and ligation of MAPCA with Blalock Taussig shunt (n = 1). The median age of the first surgery in this group was 2.4 years (0.2–14.1 years). During the follow-up, one patient had occlusion of left unifocalization graft; a further surgical operation could not be done, and she was placed in conservative palliation strategy. Two patients underwent complete repair at the age of 10.3 and 11.8 years. The first patient had left unifocalization and total correction using a 18 mm Contegra bovine valve conduit at the same stage. Postoperative course was uneventful. Cardiac catheteriation at 2 years post-procedure showed non-stenotic pulmonary arterial branches and mildly elevated pulmonary pressure of 28–30 mm Hg. The second patient had delayed sternal closure and required a cadaveric bone graft for sternal closure. This boy had residual hypoplastic right pulmonary artery, requiring serial catheter pulmonary arterial rehabilitation.

### Survival

**Figure 2:**
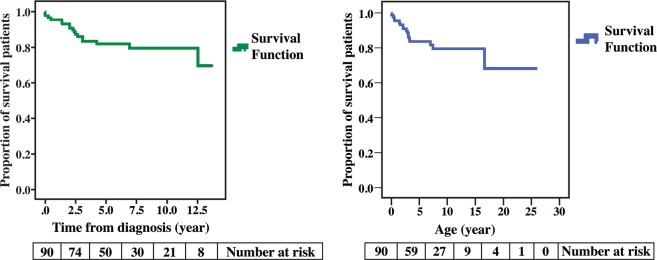
Cumulative survival rate of patients with PA/VSD (n = 90). Left panel illustrates time from diagnosis and right panel illustrates time from date of birth (age). PA/VSD: pulmonary atresia with ventricular septal defect.

At the median follow-up time of 5.7 years (7 days–13.7 years), 17 patients died from cardiac adverse events with or without other morbidities, which accounted for a mortality rate of 18.9%. Overall, the survival of patients with PA/VSD following diagnosis at 1, 5, and 10 years was 95.6%, 82.1%, and 79.6%, respectively (Fig. 2, left panel). In other words, survival of patients with PA/VSD at the age of 1, 5, and 10 years was 95.6%, 83.7%, and 79.6%, respectively (Fig. 2; right panel). Of the 17 mortality cases, 9 patients who were categorized in group 1 died from pneumonia and sepsis (n = 5) and severe hypoxemia due to possible inadequate aorto-pulmonary shunt or shunt occlusion (n = 4). Of the 6 patients categorized in group 3, 1 patient died due to sudden cyanosis and cardiac arrest at home, 2 patients had pneumonia and pulmonary hemorrhage, 2 patients had cardiac arrest early post-unifocalization and multi-organ failure. Another patient, a 16-year-old boy who had undergone complete repair at 14 years of age, died due to progressive right ventricular failure, pulmonary hypertension, and ultimately, thrombus formation in the right atrium, surgical graft and infective endocarditis at the VSD patch. In group 4, 2 patients died: 1 patient died due to pneumonia, sepsis, and pulmonary hemorrhage and the other patient died from cardiac arrest immediate to post-operative right unifocalization.

Table 2 illustrates clinical outcomes at the recent follow-up of patients in the cohort. Most patients who underwent complete repair had full oxygen saturation post-operatively and stayed NYHA functional class I. Three patients had functional class decline to III-IV within 1–3 years following total corrrection. One was a 16-year-old boy with PA/VSD, non-confluent PAs and MAPCAs who died 2.5 years post repair, as previously described. One patient was an 8-year-old boy with PA/VSD, non-confluent PAs and MAPCAs (group 4) who underwent complete repair at 7 years of age, after traditional staged right and left unifocalization. The patient had signs of right heart failure post-operatively due to pulmonary hypertension and massive thrombus in right pulmonary artery. Thrombectomy and pulmonary arterioplasty was performed; however, chronic thrombo-embolism associated with pulmonary hypertension was not resolved. He was placed on anti-coagulants and dual pulmonary vasodilators (sildenafil and bosentan). The latter patient who had declined in his functional class to III-IV post total correction was a 15-year-old boy with PA/VSD, confluent PAs, MAPCAs (group 2) who underwent complete repair at 10 years of age. This patient had postoperative pulmonary hypertension and developed signs of right sided heart failure at 1.5 years post-correction even though sildenafil was given. Cardiac catheterization was performed and showed no pulmonary stenosis or occlusion. Dual pulmonary vasodilator therapy was given and his symptoms were alleviated. These two patients survived in an NYHA functional class III.

**Table 2:** Clinical outcomes at the recent follow up (n = 90).

	Overall (N = 90)	Total repair (N = 32)	Not total repair yet (N = 58)
Age (years)	6.7 (0.05–26)	12.3 (2.83–23.02)	4.8 (0.05–26)
Time from diagnosis (years)	5.7 (0.02–13.68)	11.3 (2.82–13.75)	4.1 (0.02–12.13)
Mortality	17 (18.9%)	1 (3.1%)	16 (27.6%)
NYHA Functional class III–IV	19 (21.1%)	3 (9.3%)	16 (27.6%)

Data represented by median (range) and number (% within column).

F/U, follow up; NYHA, New York Heart Association.

**Figure 3:**
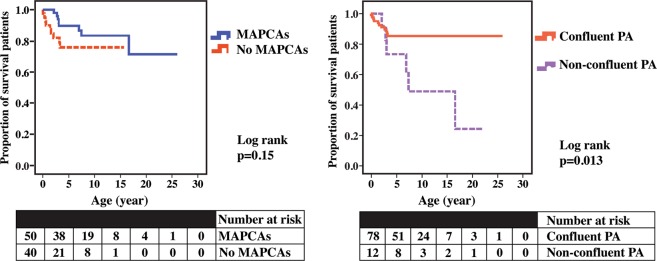
Left panel: survival rate curves of patients who had pulmonary atresia, ventricular septal defect (PA/VSD) with major aortopulmonary collateral arteries (MAPCAs): blue line; and without MAPCAs: red line. Right panel: survival rate curves of PA/VSD patients with confluent pulmonary arteries (PAs): pink line; and non-confluent Pas: purple line.

The survival rate of patients with MAPCAs at age 1, 5, and 10 years was 100%, 89.7%, and 83.3%, respectively. No statistical difference was seen in comparison to the survival curve of patients without MAPCAs, who had survival rates at age 1, 5, and 10 years of 90%, 75.9%, and 75.9%, respectively (Log-rank 0.15) (Fig. 3, left panel). With regards to the presence of confluent PAs, patients with non-confluent PAs had survival rates of 100%, 73.3%, and 48.9%, at 1, 5, and 10 years of age, respectively. This was significantly worse than the survival rates of patients who had confluent PAs with survival rates of 94.9%, 85.5%, and 85.3%, at 1, 5, and 10 years of age, respectively (Log-rank 0.013) (Fig. 3, right panel).

### Mortality risks

The patients’ characteristics were reviewed to evaluate their mortality risks (Table 3). A multivariate analysis identified two predictors of death: presence of non-confluent PAs (hazard ratio 8.09, 95% CI 1.89–34.40, *p*-value = 0.003) and presence of associated anomalies (hazard ratio 6.50, 95% CI 1.87–22.60, *p*-value = 0.002).

**Table 3:** Predictors of overall mortality (n = 90).

Factors	Crude HR (95%CI)	*p*-value	Adjusted HR (95%CI)	*p*-value
Male gender	2.78 (0.92–8.35)	0.062		
Birth weight <2,500 g	1.75 (0.58–5.23)	0.314		
Presence of genetic disorder	1.07 (0.11–10.3)	0.948		
Presence of associated anomalies	5.19 (1.68–16.0)	0.002*	6.50 (1.87–22.6)	0.01*
Oxygen saturation at first presentation <80%	1.91 (0.66–5.55)	0.227		
Presence of MAPCAs	0.65 (0.23–1.89)	0.434		
Presence of pulmonary non-confluence	6.09 (1.66–22.32)	0.003*	8.09 (1.89–34.4)	0.003*
Age at first diagnosis <1 year	3.91 (0.82–18.42)	0.068		
Age at first surgery <1 year	1.97 (0.65–5.99)	0.228		

Univariate analysis by Chi square test, Fisher’s exact test and multivariate analysis by cox regression.

Statistical significant at *p*-value < 0.05.

HR, hazard ratio; MAPCAs, major aorto-pulmonary collateral arteries; PAs, pulmonary arteries.

## Discussion

The present study used a 12-year congenital heart disease database from a large referral cardiac center in Thailand to assess contemporary outcomes of 90 children with PA/VSD. Complex PA/VSD was identified in one-third of the patients. Thirty-two patients in a cohort achieved the stage of complete repair. In a median follow-up time of 5.7 years (7 days–13.7 years), 17 patients died from any cause in addition to their severe cardiac pathophysiology, accounting for a mortality rate of 18.9%. Overall survival rates of PA/VSD at 1, 5, and 10 years of age, were 95%, 83.7%, and 79.6%, respectively. In this study, the significant predictors of death were the presence of associated anomalies and non-confluent PAs. To the best of our knowledge, this is the first retrospective cohort study reporting survival of children with PA/VSD in developing countries.

Overall survival outcomes of patients with PA/VSD have been previously reported. In a retrospective review of 129 babies born with pulmonary atresia, including PA/intact ventricular septum (PA/IVS), PA/VSD, complex PA in 1980–1995 by Leonard and colleagues^12^, the authors reported that mortality in the first year of life was 49/129 (38%), with 15/29 (52%) for PA/IVS, 15/60 (25%) for PA/VSD, and 19/40 (48%) for complex PA. In a recent publication in 2016, using a nation-wide cohort of 109 PA/VSD patients born between 1970 and 2007 in Finland^10^, the authors demonstrated survival rates at 10, 20, and 30 years of 60%, 55%, and 53%, respectively, and the low survival rates of 55 palliative patients were 42%, 30%, and 20%, at 5, 10, and 20 years, respectively. The 10-year survival rate in our study (79.6%) was slightly higher, when compared to prior studies^10,12^, suggesting that progress has taken place in the strategies for treatment in the past decade. Survival curves of patients who had PA/VSD with or without MAPCAs showed no statistical difference, which is consistent with a previous report^10^.

The strategies for surgical management of PA/VSD depend on the source of the pulmonary blood supply and the pulmonary arteries; in this study, the patients were categorized into four groups. The treatment for PA/VSD, confluent Pas, which is supplied by the ductus arteriosus or group 1, is straightforward to reconstruct the pathway from the right ventricle to the pulmonary arteries^1,3^. Traditionally, a systemic to pulmonary shunt would be the first option in small infants, followed by complete repair in later life. Nevertheless, a historic rise in mortality following systemic to pulmonary shunt has been reported^13^. Recent advances in surgical intervention, anesthesia, and postoperative care have allowed the early complete repair at advanced medical centers. In addition, catheter intervention such as ductal stenting for palliative patients while waiting for a complete repair has been introduced. Ductal stenting, however, may cause some complications such as ductal spasm, stent migration, kinking of proximal pulmonary artery, or dissection^14^. At our medical center, surgical intervention is a mainstay for management. Of the 39 patients with simple PA/VSD with confluent PAs, 37 patients underwent traditional systemic to pulmonary shunt at their initial operation. The rate of shunt thrombosis was 13.5%, which is comparable to the national database from 25 congenital heart centers in the UK in 2000–2013^13^. Fifteen patients (37.5%) in our cohort achieved total correction. Of the 15, 2 patients underwent complete repair at their initial stage at 1.2 and 2.5 years of age. Surgical re-intervention was reported in one patient for left pulmonary artery arterioplasty. No mortalities were reported following complete repair in this study cohort.

In a more complex group (PA/VSD, confluent PAs with MAPCAs; group 2), the patients had various pulmonary arterial distributions. Most patients had their pulmonary blood supply mainly from the ductus arteriosus, but some segments of the lungs were perfused by MAPCAs. Operative procedures included staged unifocalization or aortopulmonary shunt with or without ligation of MAPCAs, followed by complete repair, as is the traditional procedure^4,15^. Eleven patients (55%) achieved their stage of total correction. The mean age at total correction was 10.8 ± 4.5 years, which was older than that of group 1, due to the complexity of the pulmonary artery anatomy. No redo pulmonary valve replacement or surgical arterioplasty was required in the median time of follow-up (7 years).

Management of the most complex group (PA/VSD with non-confluent PAs; group 3), with exclusive blood supply to both lungs by MAPCAs is challenging. Constructed pulmonary artery using Dacron vascular graft was performed in some unifocalization procedures in preparation for the central pulmonary artery proper prior to the next step of the operation (right ventricular to pulmonary artery conduit placement) (see Supplementary Fig. S1)^4,6^. In the cohort, 12 patients had 24 surgical interventions and 4 of these achieved complete repair. In the group, six patients (50%) died during follow-up. Accordingly, patients with non-confluent PAs in the study had survival rates of 100%, 73.3%, and 48.9%, at 1, 5, and 10 years of age. This rate is comparable to the survival rate of patients who had an initial palliative procedure in a large-scale study at Children’s Hospital Stanford^11^, which reported a 5-year actuarial survival rate of 82%. Nevertheless, this is far less than the survival rate of single-stage repair (midline unifocalization), with a 5-year survival of 95%^11^.

In a multivariate analysis, the presence of non-confluent PAs was found to be an independent mortality risk factor (hazard ratio 6.50, 95% CI 1.87–22.60, *p*-value = 0.002). This factor was found to be one of the mortality risks in 355 PA/VSD patients who had surgical complete repair in a large cohort study from the Mayo clinic^15^. The presence of confluent native PAs naturally promotes its growth by several techniques such as the modified Blalock Taussig shunt, central shunt, and catheter rehabilitation, which improves perfusion and enhances the success rate of total repair^4,11,16^. Even though diminutive and underdeveloped native PAs are filled by retrograde flow from MAPCAs (group 4), the intrapulmonary branching pattern can be much better than the non-confluent PAs and they exclusively depend on MAPCAs (group 3).

Forty-three patients in the cohort underwent a surgical procedure in infancy, while 45 patients underwent operations later in life. Of the 88 patients who underwent surgical procedures, 35% achieved complete intracardiac repair: 17/49 patients with MAPCAs and 15/39 patients without MAPCAs. The most popular conduit type used in the medical center for this cohort was the bovine jugular vein-valved conduit (Contegra; Medtronic, Inc.), because of its availability in several sizes, followed by pulmonary homograft. Larger and older patients tend to undergo repair with larger conduits. The longevity of conduits was not explored in this cohort, because no re-operation for conduit failure was performed and the follow-up time to be assessed was likely too short. In the literature, many reports indicate that conduit size has a crucial influence on conduit longevity: the smaller the conduit diameter, the shorter the longevity^3,16,17^. In addition, postoperative pulmonary artery pressure has been noted to be reversely correlated to conduit longevity in patients with PA/VSD with MAPCAs^16^.

We also found that associated anomaly is another predictor of death (hazard ratio 6.50, 95% CI 1.87–22.60, *p*-value = 0.002). Genetic syndromes, VACTERL association, and kidney agenesis were included among the associated anomalies. The 22q11.2 microdeletion syndrome, reported in 30–40% of patients with PA/VSD in prior publications^11,18^, was less frequently found in our study (4.4%). Oxygen saturation at the first presentation, birth weight, diagnosed in infancy, age at first operation less than one year of age, and presence of MAPCAs were not associated with mortality. In comparison, the study from the Hospital for Sick Children, Toronto^19^ that included 118 patients with simple PA/VSD and 53 patients with PA/VSD-MAPCAs, younger age at repair, earlier birth cohort, fewer bronchopulmonary segments supplied by native pulmonary arteries, and initial placement of a systemic-pulmonary artery shunt were found to be independent risk factors of death after initial surgery. Cho and coworkers^15^ demonstrated that the independent mortality risk in 160 PA/VSD patients who had palliative surgery included presence of MAPCAs, while male sex, non-confluent pulmonary arteries, reopening of the ventricular septal defect, and post-repair conduit exchange were defined as mortality predictors in 355 patients who had complete repair of PA/VSD.

### Study limitations

As this is a retrospective-cohort and observational study at a single medical center, selective bias is inevitable. The authors therefore strictly selected children with PA/VSD who had confirmed diagnoses of PA/VSD at the center, and accounted for incident cases. Although surgical lists of patients for PA/VSD repair prior to 2005 were available and they could have been counted as prevalent cases, to eliminate bias, the authors only included patients from 2005 to 2016 due to the incomplete hospital database of incident cases prior to 2005. A limitation of incomplete genetic testing was also noted in the earlier era. Of the 90 PA/VSD patients, 80 patients had a known status by the end of 2018. Re-operation, redo pulmonary valve replacement has not been performed during the cohort study. Another major aspect that was not covered in this report is the burden of catheter re-intervention, which may be included in aggregated data in our future research.

## Conclusion

Children with PA/VSD have had a fairly good survival rate for the past decade. Overall survival rates for PA/VSD at 1, 5, and 10 years of age were 95%, 83.7%, and 79.6%, respectively. Despite multi-stage interventions, some patients can achieve operative correction. In our experience, traditional staged unifocalization followed by complete repair in PA/VSD and MAPCAs yields a modest survival rate, especially in patients with confluent PAs. Significant predictors of death were presence of associated anomalies and non-confluent PAs. The deliberative evaluation of anatomy and source of pulmonary blood supply is required to manage this lesion. Long-term and aggregated data will also be beneficial to arrive at the optimal treatment and outcomes of simple and complex PA in Thailand.

## Materials and Methods

The present study was a single-center, observational study using a hospital database from a large referral cardiac center in Thailand. Following the approval of the Siriraj Institutional Review Board Faculty of Medicine Siriraj Hospital, Mahidol University, all incident patients who were diagnosed with PA/VSD by echocardiography between January 1, 2005 and December 31, 2016 in Siriraj Hospital, Mahidol University, were retrospectively reviewed. Patients with single ventricle or those who had undergone a cardiac operation at another hospital were excluded. The requirement of informed consent from patients was waived and the process for protecting patient confidentiality was guaranteed. Permission for the study protocol to waive the informed consent process was approved by Siriraj Institutional Review Board Faculty of Medicine Siriraj Hospital, Mahidol University [Study number 212/2560 (EC1)]. Demographic data was collected for age at referral and diagnosis, birth weight, gender, oxygen saturation, associated anomalies, genetic disorder, initial presentation, and cardiac findings including presence of confluent pulmonary artery, presence of MAPCAs, and size of pulmonary arteries. As the strategies for surgical management of PA/VSD depend on the initial anatomy of pulmonary arteries, the source of pulmonary blood supply, patients were categorized into four groups: (1) PA/VSD with confluent PAs, (2) PA/VSD, confluent PAs, MAPCAs, (3) PA/VSD, non-confluent PAs, MAPCAs, and (4) PA/VSD, small native PAs, MAPCAs. Surgical interventions and clinical outcomes were recorded including functional class, oxygen saturation, right heart failure, and mortality following diagnosis at the most recent follow-up at the end of 2018.

### Statistical analysis

The patients’ baseline characteristics and outcomes were summarized using descriptive statistics. Normally distributed data was presented as mean ± SD, while the median with range was used where the distribution of data was not normal. Categorical data was represented as a number and a percentage (%). Differences between the categorical data were assessed using a Chi-square. Cumulative survival, from date of birth and date of diagnosis to the mortality endpoint, was calculated using the Kaplan-Meire analysis with log-rank test. The relation between baseline characteristics and mortality was evaluated with Cox regression and multivariate analysis. A *p*-value < 0.05 was considered to be statistically significant. The statistical analyses were performed with SPSS 20.0 for Windows (SPSS Inc., Chicago, IL, USA).

## Supplementary information

Supplementary dataset.

## Data Availability

The datasets generated during and/or analyzed during the current study are not publicly available due to patient confidentiality, but they are available from the corresponding author and Siriraj institutional committee on reasonable request.
