# Supplementary material for: Current era outcomes of pulmonary atresia with ventricular septal defect: A single center cohort in Thailand
Source: Sci Rep. 2020 Mar 20;10:5165. doi: 10.1038/s41598-020-61879-2 (PMC7083910; doi:10.1038/s41598-020-61879-2)
Supplement: Supplementary file 1 — Supplementary dataset. [file 41598_2020_61879_MOESM1_ESM.docx]

**Current era outcomes of pulmonary atresia with ventricular septal defect: A single center cohort in Thailand**

Kanthalas Lertsakulpiriya MD^1+^, Chodchanok Vijarnsorn MD^1*^, Prakul Chanthong MD^1^, Paweena Chungsomprasong MD^1^, Supaluck Kanjanauthai MD^1^, Kritvikrom Durongpisitkul MD^1^, Jarupim Soongswang MD^1+^, Thaworn Subtaweesin MD^2+^, Somchai Sriyoschati MD^2+^

^1^ Department of Pediatrics, Faculty of Medicine Siriraj Hospital, Mahidol University, Bangkok, Thailand

^2^ Department of Surgery, Faculty of Medicine Siriraj Hospital, Mahidol University, Bangkok, Thailand

**Corresponding Author:**

Chodchanok Vijarnsorn MD

Department of Pediatrics, Faculty of Medicine Siriraj Hospital, Mahidol University

2 Wanglang Rd., Bangkok, Thailand 10700

Tel 011-66-2-4197000 ext 5672

Fax 011-66-2-4195960

Email: [cvijarnsorn@yahoo.com](mailto:cvijarnsorn@yahoo.com)

**Supplementary data**

**Supplementary Table S1.** Surgical management for PA/VSD in a cohort (n=90)

|  | Complete repair  (n=32) | During staged operation (n=56) | No operation performed (N=2) |
| --- | --- | --- | --- |
| MAPCAs  - Present  - Absent | 17 (53.1%)  15 (46.9%) | 32 (57.1%)  24 (42.9%) | 1 (50%)  1 (50%) |
| Confluent PAs  - Present  - Absent | 28 (87.5%)  4 (12.5%) | 48 (85.7%)  8 (14.3%) | 2 (100%)  0 (0%) |

Data represented by n (% within column)

MAPCAs, major aorto-pulmonary collateral arteries; PAs, pulmonary arteries.

**Supplementary Fig. S1**

**
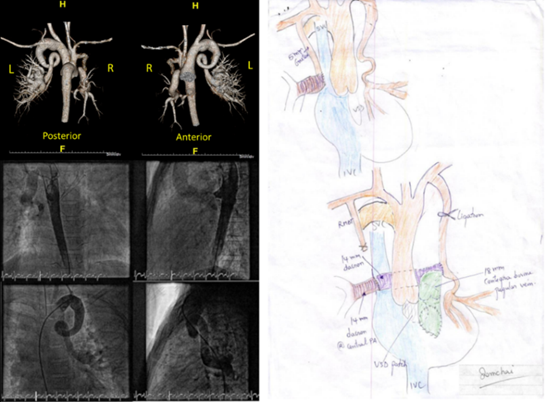
**

**Supplementary Figure S1**. Left panel: pre-operative computerized tomographic angiography and cardiac angiography of patient with pulmonary atresia, ventricular septal defect (PA/VSD), and non-confluent pulmonary arteries supplied by major aortopulmonary collateral arteries (MAPCAs) who had undergone right unifocalization, constructed central right pulmonary artery using Goretex graft and the left lung perfused by tortuous large MAPCA from left subclavian arteries. Right panel: diagram of surgical operation of total correction using 18 mm Contegra bovine jugular conduit and ligation of residual left MAPCAs
